# Estimation of Tooth Size Discrepancies among Different Malocclusion Groups

**DOI:** 10.5005/jp-journals-10005-1242

**Published:** 2014-08-29

**Authors:** Narender Hasija, Madhu Bala, Virender Goyal

**Affiliations:** Professor, Department of Pedodontics, JCD Dental College, Sirsa Haryana, India; Reader, Department of Pedodontics, JCD Dental College, Sirsa Haryana, India; Professor, Department of Pedodontics, Dasmesh Institute of Research and Dental Sciences, Faridkot, Punjab, India

**Keywords:** Bolton, Tooth size, Anterior ratio, Overall ratio

## Abstract

**Regards and Tribute:** Late Dr Narender Hasija was a mentor and visionary in the light of knowledge and experience. We pay our regards with deepest gratitude to the departed soul to rest in peace.

Bolton’s ratios help in estimating overbite, overjet relationships, the effects of contemplated extractions on posterior occlusion, incisor relationships and identification of occlusal misfit produced by tooth size discrepancies.

**Aim:** To determine any difference in tooth size discrepancy in anterior as well as overall ratio in different malocclusions and comparison with Bolton’s study.

**Materials and methods:** After measuring the teeth on all 100 patients, Bolton’s analysis was performed. Results were compared with Bolton’s means and standard deviations. The results were also subjected to statistical analysis. Results show that the mean and standard deviations of ideal occlusion cases are comparable with those Bolton but, when the mean and standard deviation of malocclusion groups are compared with those of Bolton, the values of standard deviation are higher, though the mean is comparable.

**How to cite this article:** Hasija N, Bala M, Goyal V. Estimation of Tooth Size Discrepancies among Different Malocclusion Groups. Int J Clin Pediatr Dent 2014;7(2):82-85.

## INTRODUCTION

Advances in the diagnostic phase of the treatment have been plentiful, particularly wrt the use of cephalometric headfilms as a pretreatment guide. The frequent use of these aids has caused us to ignore one of the most basic fundamentals, i.e. tooth size. The term tooth size particularly refers to the mesiodistal width of the tooth. Every single tooth size discrepancy can be troublesome and their accumulation along the arch can produce difficulties in achieving perfect occlusion. Although, the natural teeth match very well in most individuals, approximately 5% of the population has some degree of disproportion among the sizes of individual teeth.^1^ It is very common to achieve a perfect class I molar relationship and yet not be able to achieve a similar cuspid relation because of tooth size discrepancies.

Various studies have attempted to assess the tooth size discrepancies *viz* Black, Bolton, Ballard, Lundstrom, etc.^2-4,6^ Bolton^3^ in 1958 measured mesiodistal width of 12 maxillary teeth first molar of one side to the first molar of the opposite side and compared with the sum derived by the same procedure carried out on 12 mandibular teeth. He found a nter ior a nd over all rat ios for tooth size. Bolton’s ratios help in estimating overbite, overjet relationships, the effects of contemplated extractions on posterior occlusion, incisor relationships and identification of occlusal misfit produced by tooth size discrepancies. The present study was done to compare tooth size ratios of the study sample with Bolton study and comparison of tooth size discrepancy in different malocclusions.

## REVIEW OF LITERATURE

Black was one of the first persons who measured a large number of human teeth and prepared tables for average mesiodistal widths of various teeth .^2^ Bolton WA stated that a correct maxillary and mandibular mesiodistal tooth size relationship is important to achieve proper occlusal interdigitation in the finishing stages of orthodontic treatment. He concluded that an overall ratio of 91.3 and an anterior ratio of 77.2 were necessary for proper coordination of maxillary and mandibular teeth.^3^ Ballard ML measured 500 sets of casts and compared the mesiodistal diameters of each tooth on one side of the dental arch with the opposite side. Ninety percent of the sample demonstrated a right left discrepancy amounting to 0.25 mm or more.^4^ Neff (1949) found that the ratio of anterior teeth size is mathematically related to overbite, determined the coefficient of the anterior teeth.^5^ Lundstrom^6^ studied the tooth size ratio between maxillary and mandibular anterior teeth, which he called ‘the anterior index’. Carey published a method of analysis for estimation of sizes of lower cuspid and bicuspid by measuring the mesiodistal diameter of lower four incisors.^7^

## MATERIALS AND METHODS

The present st udy was conducted on dent al ca sts of 100 individuals selected from Outpatient Department of Pedodontics and P reventive Dentist r y, Dental College, Roht ak (Haryana).

### Criteria for Selection

- Good quality pretreatment models of maxillary and mandibular arch.
- History of no extractions or any type of proximal stripping.
- Presence of all erupted permanent teeth.

Hundred patients were divided into four groups as follows:

1. Class I – 25
2. Class II division 1 – 25
3. Class II division 2 – 25
4. Class III – 25

**Fig. 1:**
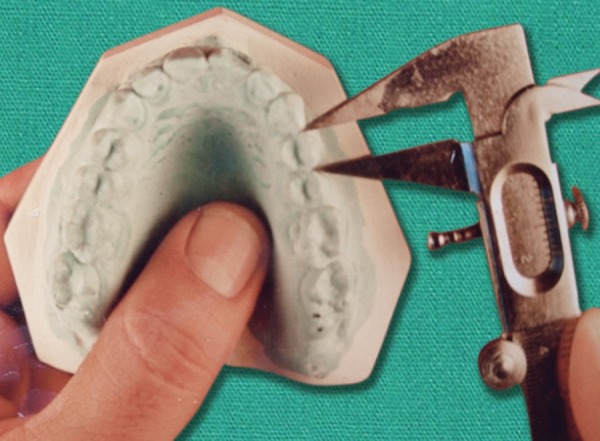
Measurement at contact points on proximal surface parallel to occlusal surface

**Fig. 2:**
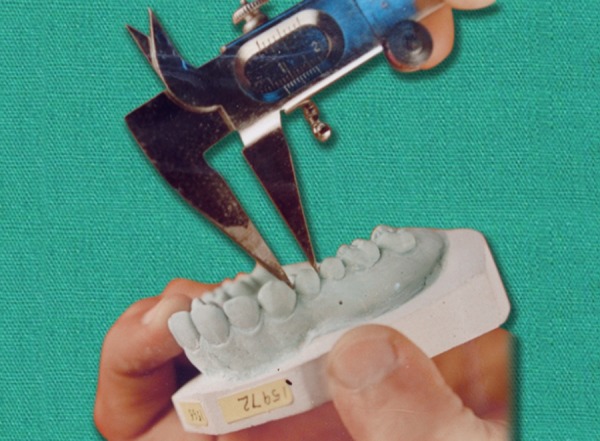
Measurement at contact points on proximal surface parallel to vestibular surface

Each and every tooth on each cast was measured at the contact point for the mesiodistal diameter with the help of Vernier Calipers accurate to 0.1 mm (Figs 1 and 2). After measuring the teeth on all 100 patients, Bolton’s analysis was performed. Results were compared with Bolton’s means and standard deviations. The results were also subjected to statistical analysis. The mean, standard deviation and standard error values were calculated.

## RESULTS

Each malocclusion has be en obse r ved i nde pendently. W hen the means and standard deviations of Bolton study are applied to present study for the anterior and overall ratios. Some patients measurement fall outside 2SD. In class 1 malocclusion group, three patients have anterior ratios greater than two standard deviation (referring to Bolton’s standard deviation) above Bolton’s means. Within the same group, three patients have anterior ratio greater than two standard deviations below Bolton’s mean. Therefore, six patients of class 1 malocclusion group have anterior ratio greater than two standard deviations from Bolton’s mean. Also class 1 malocclusion group has two patients with overall ratios greater than two standard deviations above the mean, and three patients have overall ratios greater than two standard deviations below the mean. Therefore, five patients have overall ratios greater than two standard deviations.

In class 2 division 1 group, three patients have anterior ratio greater than two standard deviations from Bolton’s mean and two patients have anterior ratio greater than two standard deviations below the mean. Also, one patient of this group shows overall ratio greater than two standard deviations above Bolton’s mean and one patient has overall ratio greater than two standard deviations below Bolton’s mean.

In class 2 division 2 malocclusion group, five patients have anterior ratio greater than two standard deviations above Bolton’s mean, and two patients have anterior ratio greater than two standard deviations below Bolton’s mean. Also, two patients have overall ratios greater than two standard deviations above Bolton’s mean, and one patient has overall ratio greater than two standard deviations below Bolton’s mean.

In class 3 malocclusion, five patients have anterior ratio greater than two standard deviations above Bolton’s mean and three patients have anterior ratio greater than two standard deviations below the mean. Also, two patients of this group show overall ratio greater than two standard deviations above Bolton’s mean, and three patients have overall ratio greater than two standard deviations below Bolton’s mean.

**Graph 1:**
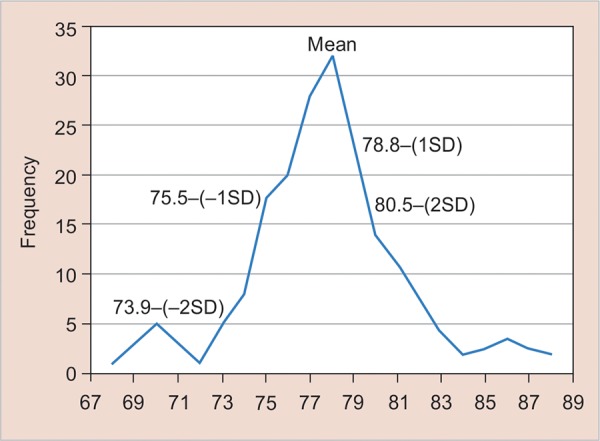
Mesiodistal dimension (anterior ratio of total sample)

**Graph 2:**
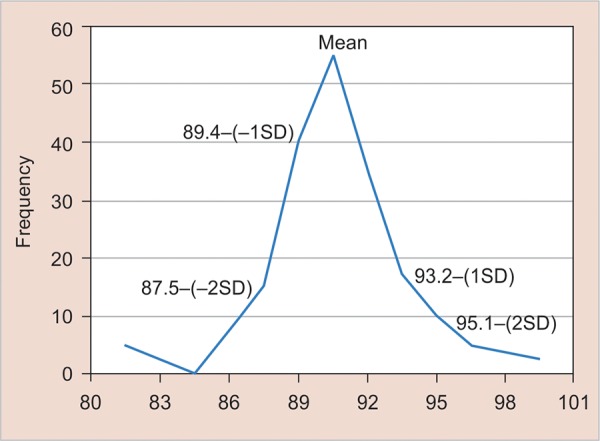
Mesiodistal dimension (overall ratio of total sample)

When the total sample is considered, 16 patients have anterior ratio greater than two standard deviations above Bolton’s mean and 10 patients have anterior ratio greater than two standard deviations below Bolton’s mean (Graph 1). Also, 7% patients have overall ratio greater than two standard deviations above Bolton’s mean and 8% patients have overall ratio greater than two standard deviations below Bolton’s mean (Graph 2).

## DISCUSSION

Bolton in 1958 established the values for intermaxillary tooth size ratios for anterior (77. 20) as well as over all (91.3 0) segment of teeth by using a sample size of 55 cases with ideal occlusion.^3^ Crosby and Alexander^8^ found that means of intermaxillary tooth size ratios in different malocclusion groups were comparable to Bolton’s means, whereas the standard deviations were higher than those of Bolton’s.

In the present study, where the combined sample of simple and malocclusion groups has been taken the results show that the mean and standard deviations of ideal occlusion cases are comparable with those Bolton but when the mean and the standard deviation of malocclusion groups are compared with those of Bolton, the values of standard deviation are hig her, thoug h the mean is compa r able. T his f ndi ng is in conformity with the study of Crosby and Alexander.^8^

Fattahi et al revealed that the mean anterior ratio (79.01) for the whole sample was statistically significantly different from Bolton’s (77.20) but no significant difference was found for the overall ratio.^9^ Toshiya et al revealed no significant differences in anterior or overall ratios among the malocclusion groups.^10^ Batool et al found significantly higher mean anterior tooth ratios for class II (p < 0.01) patients. A ll othe r ratios were w ith in close range of Bolt on’s nor ms.^11^ According to Lopatiene, comparison of overall and anterior Bolton’s ratio revealed no statistically significant difference between Angle class I, II and III.^12^ Al Khateeb and Abu Alhaija^13^ in 2006 found no statistically significant differences in Bolton’s ratios between the different malocclusions. Their sample consisted of 140 orthodontic models of school children aged between 13 and 15 years of Jordanian origin.

A number of cases within each malocclusion group have ratios greater than two standard deviations from Bolton’s mean.^3^ This is in accordance with study by Crosby and Alexander.^8^ According to Crosby and Alexander, any figure outside two standard deviations from Bolton’s mean represent 2 to 3 mm tooth size discrepancy which must be considered clinically significant.^8^ In the present study, one out of four cases has increased anterior tooth size ratio and one in seven cases has increased overall tooth size ratio. That means more cases have increased anterior tooth size ratios. It was found that 60% among increased anterior toot h size rat io have i ncrea sed mandibu lar toot h si ze excess. According to Batool et al, skeletal class II patients showed a tendency toward higher mesiodistal widths of teeth in the mandibular anterior region.^11^

Bolton has categorically stressed that dental arches should be considered as consisting of two component, i.e. anterior and posterior and the ratio of 77.2 in the anterior segment is very specific and is completely independent of overall ratio, since the highest dispersion of standard deviation in anterior ratio as compare to overall ratio, the dispersion of value is far less in posterior segment (Tables 1 to 3).^3^

The average mesiodistal measurements for maxillary canine and mandibular lateral incisor fall in the higher range of values than the average values of corresponding teeth by Black and Ballard.^2,4^ The discrepancy was only present in malocclusion groups and not in ideal occlusion.

## CONCLUSION

The values of means of intermaxillary tooth size ratio are comparable in ideal occlusion cases and different malocclusion groups; therefore, it can be concluded that Bolton’s analysis is applicable to all cases irrespective of type of malocclusion and remains are essential investigation before starting the treatment for post-treatment stability of arches.

**Table 1:** **Table 1:** Anterior ratio

*S. no.*		*Malocclusion*		*Mean*		*Range*		*SD*		*SE*		*t-value (with Bolton)*	
1		Class 1		78.04		68.72-87.20		4.39		0.88		0.763	
2		Class 2 div. 1		77.89		70.23-84.76		2.93		0.58		0.862	
3		Class 2 div. 2		78.08		69.37-87.35		3.80		0.76		0.897	
4		Class 3		77.41		69.30-86.71		3.83		0.77		0.212	
5		Total sample		77.85		69.30-87.35		3.68		0.37		1.101	

**Table 2:** **Table 2:** Overall ratio

*S. no.*		*Malocclusion*		*Mean*		*Range*		*SD*		*SE*		*t-value (with Bolton)*	
1		Class 1		90.50		82.03-98.03		3.81		0.76		0.784	
2		Class 2 div. 1		90.45		82.87-96.09		2.73		0.55		1.049	
3		Class 2 div. 2		90.61		81.47-96.85		2.94		0.59		0.811	
4		Class 3		90.54		82.64-96.27		2.73		0.55		0.938	
5		Total sample		90.53		81.47-98.37		3.13		0.31		1.350	

**Table 3:** **Table 3:** Anterior ratio and overall ratio

		*N*		*Anterior ratio*								*Overall ratio*							
				*Mean*		*Range*		*SD*		*SE*		*Mean*		*Range*		*SD*		*SE*	
Bolton		55		77.20		74.5-80.4		1.65		0.22		91.30		87.5-95.8		1.91		0.26	
X		100		77.85		69.30-87.35		3.68		0.37		90.53		81.47-98.37		3.13		0.31	
